# Longitudinal Ultrasound Assessment of Achilles and Patellar Tendon Morphology in National Collegiate Athletic Association Division 1 Female Gymnasts

**DOI:** 10.3390/jfmk11020185

**Published:** 2026-05-05

**Authors:** Phillip Hartog, Lee J. Hinkle, Ulrike H. Mitchell, Aaron Wayne Johnson

**Affiliations:** 1 Department of Exercise Sciences, Brigham Young University, Provo, UT 84602, USA; phillip_hartog@byu.edu (P.H.);; 2 Neuromuscular Physiology Lab, Department of Kinesiology, Kansas State University, Manhattan, KS 66506, USA

**Keywords:** soft tissue adaptation, sonographic imaging, lower extremity structures, athletic load response, musculoskeletal screening, overuse injury risk

## Abstract

**Background:** Collegiate gymnastics imposes high repetitive loads on the lower extremities, particularly the Achilles and patellar tendons, yet longitudinal data describing tendon adaptation across a competitive season remain limited. **Objectives:** To examine seasonal changes in Achilles and patellar tendon morphology (thickness, cross-sectional area [CSA], echogenicity, vascularity, and symmetry) across a twelve-month competitive cycle in Division I female gymnasts and to explore relationships with pain. **Methods:** This longitudinal observational study included twenty-five Division I female gymnasts (age: 20.0 ± 1.6 years; height: 159.5 ± 6.2 cm; weight: 57.8 ± 5.7 kg). Bilateral ultrasound assessments of the Achilles and patellar tendons were performed at three time points (post-summer, preseason, and postseason). Tendon thickness, CSA, echogenicity, and vascularity were evaluated using standardized imaging protocols. Symmetry indices were calculated, and pain was assessed using validated scales. Normality was assessed using appropriate statistical tests. Parametric data were expressed as mean ± standard deviation (SD), and non-parametric data as median and interquartile range. Paired comparisons were conducted using paired *t*-tests or Wilcoxon signed-rank tests, with Holm correction applied for multiple comparisons. **Results:** Achilles tendon thickness increased from summer to postseason (*p* < 0.05), with no significant changes in CSA after adjustment. Echogenicity and vascularity remained unchanged. Patellar tendon morphology was largely stable; however, left proximal thickness decreased from summer to preseason and remained reduced at postseason (*p* < 0.01), with no other consistent regional changes. Pain prevalence increased modestly across the season without a clear lateralized pattern or association with symmetry indices. **Conclusions:** Achilles tendon thickness appears to be a sensitive marker of seasonal adaptation in female collegiate gymnasts, whereas patellar tendon morphology remains stable. These findings support the use of longitudinal ultrasound monitoring for athlete screening and load management.

## 1. Introduction

Gymnastics is a highly demanding sport that requires exceptional levels of strength, flexibility, coordination, balance, and artistry to perform complex, acrobatic skills across multiple apparatuses [1,2,3,4]. For example, the apparatus for female collegiate gymnastics includes the vault, uneven bars, balance beam, and floor. Each apparatus imposes unique mechanical and technical demands that necessitate precise motor control [4]. Over time, gymnastics has evolved from its origins in ancient physical conditioning practices into a globally recognized, elite sport characterized by early specialization, intensive year-round training, and high physical and psychological demands [4]. These factors potentially lead to pain [5,6], injuries [1,2,3,4], missed practices [4,7] or participation in competitions [2,4,7].

During training and competition, gymnasts experience repetitive, high-impact loading, particularly during tumbling, vaulting, and plyometric maneuvers such as jumps and leaps on the balance beam [1,2,3,4]. These actions impose substantial mechanical stress on the lower extremities, especially on the Achilles tendons, which can experience repetitive loads of up to 15 times a gymnast’s body weight during take-off and landings [2,3,4].

From an exercise physiology standpoint, tendon adaptation depends on how much load is applied, how often it occurs, and how much recovery time is allowed. Proper load management, along with eccentric and progressive training, can strengthen tendons and lower the risk of injury [5,6]. Given the early onset and cumulative nature of training in gymnastics, athletes are exposed to repetitive mechanical loads during critical periods of musculoskeletal development, predisposing them to overuse and degenerative tendon conditions [2,5,8]. Tendon injuries involve collagen disruption, matrix breakdown, and altered cellular signaling. Repeated loading can upset the balance between repair and breakdown, leading to thicker, less stiff, and potentially degenerated tendons [5,6,9].

Tendon morphology and ultrasound appearance provide complementary, but not definitive, information about tendon response to loading. Vascularity, echogenicity, thickness, and cross-sectional area (CSA) each reflect different aspects of tendon structure and potential remodeling. Reduced echogenicity may indicate altered fibrillar organization, increased ground substance, or localized edema, although abnormal ultrasound findings can also occur in asymptomatic athletes and do not consistently correspond with pain or functional limitation [5,10,11,12,13]. Similarly, vascularity alone is not a reliable indicator of tendon pathology, as mechanical loading, collagen organization, and regional stress distribution also influence tendon health [1,9,10]. Tendon thickness and CSA provide quantitative measures of morphology, but they should be interpreted cautiously because increased tendon size may reflect either load-related remodeling or pathological swelling depending on the broader clinical and imaging context [5,10,11,12,13,14,15].

Although clinical examination remains an essential first step in evaluating tendon pathology, it is often limited by subjective interpretation and can fail to detect subclinical or early structural changes. Musculoskeletal ultrasound provides valuable diagnostic advantages for visualizing fiber alignment, vascular changes, and tendon size which are not discernible through palpation or functional testing alone [11,12,13,14,15]. Incorporating imaging into clinical assessment enhances diagnostic accuracy, supports individualized rehabilitation planning, and allows for longitudinal monitoring of tendon adaptation or recovery.

Despite the high prevalence of tendon-related pathology in gymnastics, there remains a clear gap in longitudinal imaging studies examining how Achilles and patellar tendon morphology change across a competitive season in collegiate female gymnasts [8,14,16]. Most existing studies are cross-sectional or focused on younger athletes, limiting understanding of whether observed tendon morphology reflects seasonal load response, chronic adaptation, or early structural concern in collegiate gymnasts [13]. Previous ultrasound studies in elite gymnasts have demonstrated tendon structural differences compared with non-athletic controls, including increased thickness and altered echotexture, even in the absence of symptoms; however, these findings are primarily derived from cross-sectional designs and may not reflect longitudinal adaptation in collegiate populations [13]. Addressing this gap may improve interpretations of ultrasound findings in a population exposed to repeated high-impact loading throughout the training and competition cycle.

Therefore, the primary aim of this study was to assess seasonal changes in Achilles and patellar tendon morphology, including CSA, thickness, echogenicity, vascularity, and symmetry, across a competitive season in Division I collegiate female gymnasts. A secondary aim was to explore how these structural measures relate to pain. We hypothesized that tendon morphology would change across the season and that greater interlimb asymmetry would be associated with greater pain.

## 2. Materials and Methods

### 2.1. Participants

This study was designed as a longitudinal observational study conducted at Brigham Young University, Provo, UT, USA. Participants were recruited as a convenience sample from a single National Collegiate Athletic Association Division I gymnastics program at the same institution. Data were collected across a single competitive season. Because the study was observational and did not involve an intervention, it was not registered as a clinical trial. A formal sample size calculation was not performed; instead, our sample was a sample of convenience and all eligible team members were invited to participate.

All athletes had a minimum of 10 years of gymnastics experience and were training and competing in NCAA-sanctioned events. Gymnasts trained under NCAA regulations that limit organized athletic activities to a maximum of 20 h per week during preseason and competitive seasons, providing a standardized upper bound for training exposure. Although organized training time is regulated, training load variables such as jump counts, landings, and event-specific exposure were not directly quantified in this study. As a result, the relationship between mechanical loading and tendon adaptation cannot be explicitly determined. Individual athlete’s exposures may have varied based on event specialization, injury status, and rehabilitation demands. Athletes’ characteristics, including injury history, leg dominance (self-reported and observed), primary events (e.g., vault, bars, beam and floor), and pain prevalence, were recorded.

### 2.2. Experimental Procedures

These athletes were tracked over a gymnastics competitive season at three time points. Before data collection commenced, the study was reviewed and approved through the Brigham Young University Institutional Review Board process (IRB2024-254-BYU) on [19 August 2024]. The experimental procedures were conducted in accordance with Brigham Young University IRB standards. This study utilized preexisting data collected during routine sports medicine practice.

**Figure 1:**
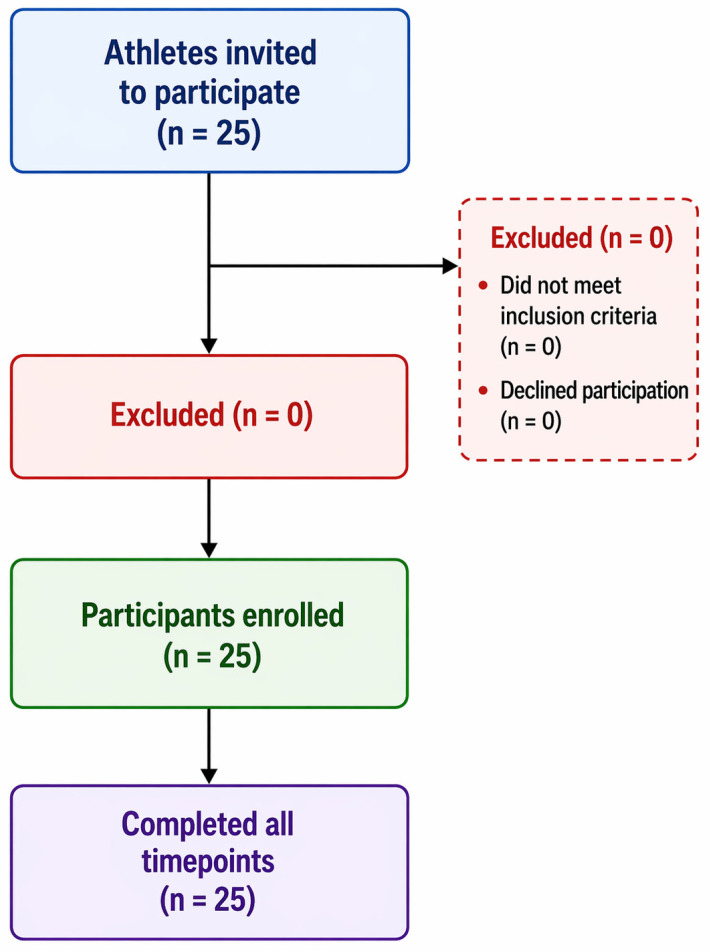
Participant flow diagram illustrating recruitment, exclusion, enrollment, and completion of study time points. A total of 25 athletes were invited, with no exclusions, and all participants completed all assessment timepoints.

Participants’ recruitment, inclusion, and retention throughout the study are illustrated in Figure 1. To assess longitudinal tendon adaptations across a competitive season, standardized ultrasound protocols were used to evaluate Achilles and patellar tendon morphology (thickness and CSA) and vascularity in Division I female collegiate gymnasts. Imaging was performed at three time points: Summer (July/August; post-summer baseline), Preseason (December), and Postseason (April). Summer and Preseason assessments were conducted immediately after full practices to reflect typical loading conditions.

**Figure 2:**
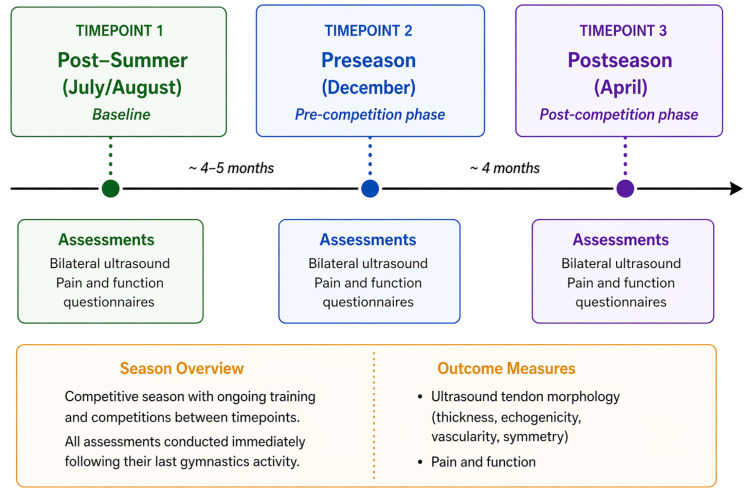
Study design and timeline of data collection. Assessments were conducted at three timepoints across the competitive season: post-summer (July/August), preseason (December), and postseason (April). At each timepoint, bilateral ultrasound imaging and pain questionnaires were performed.

Imaging was performed at three time points across the competitive season: Summer (July/August; post-summer baseline), Preseason (December), and Postseason (April). With Summer and Preseason assessments conducted immediately after practice to reflect typical loading conditions. Please see Figure 2. For more details. At Postseason, imaging was performed both before and after practice to evaluate resting tendon characteristics and acute loading effects. Symmetry indices were calculated, and validated scales were used to assess pain and function. Because Summer and Preseason imaging was conducted immediately post-practice, these measures may reflect acute tendon responses to loading rather than true structural adaptation. Although Postseason pre-practice imaging better approximates resting tendon characteristics, differentiation between acute and chronic adaptation remains limited. A uniform standard operating procedure was followed to ensure repeatability and minimize operator variability.

### 2.3. Pain Assessment

Each participant was assigned a unique identification number and completed a demographic questionnaire via Qualtrics (XM Platform 2024–2025). The Visual Analog Scale (VAS) was used to assess the intensity of pain experienced by participants following gymnastics practice [5]. This is a method that is consistent with prior longitudinal ultrasound studies in competitive gymnasts [9,12]. It consisted of a 10 cm horizontal line, anchored by two descriptors at each end representing the extremes of paint intensity: 0 being “no pain” and 10 being the “worst pain imaginable”. During data collection, participants are instructed to mark a point on the line that corresponds to their current level of pain. The participant’s mark is then measured and recorded as the VAS pain score. Pain outcomes were recorded as binary indicators (presence/absence) for the Achilles and patellar tendons based on athlete self-report. Missing pain responses were treated as missing data and excluded from analyses rather being than coded as the absence of pain to avoid systematic bias.

### 2.4. Ultrasound Imaging Protocol

**Figure 3:**
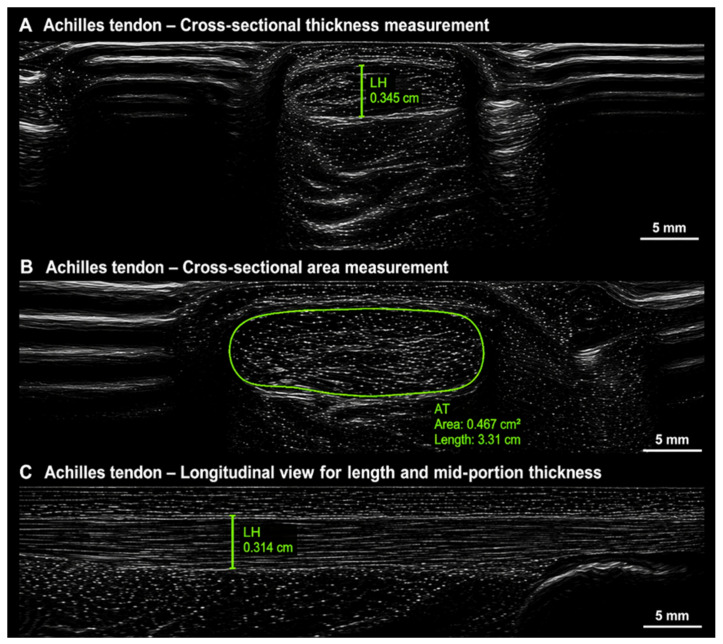
Representative ultrasound images of the Achilles tendon. (**A**) Cross-section thickness measurement at the superficial, midpoint, and deep regions. (**B**) Cross-sectional area measurement of the Achilles tendon. (**C**) Longitudinal view demonstrating tendon thickness at the mid-portion. Scale bars = 5 mm. Abbreviations: LH = lateral thickness; AT = Achilles tendon; cm = centimeters; cm^2^ = square centimeters.

Achilles tendon imaging was performed using a FORTIS ultrasound system equipped with an ML6–15 linear transducer (GE HealthCare, Chicago, IL, USA). Imaging parameters were standardized across all participants with a depth of 2.0 cm, frequency of 15 MHz, and gain of 56. Participants were positioned prone on a treatment table with the ankles dorsiflexed to 90°, maintained manually or with external support. The intermalleolar line was identified by palpating and marking the medial and lateral malleoli and bisecting the line to establish a consistent midpoint for probe placement. Bilateral imaging was conducted using a standardized sequence. Short-axis imaging was performed at the intermalleolar midpoint with cineloop acquisition, followed by long-axis imaging centered over the same midpoint to obtain a longitudinal tendon image. Panoramic imaging (LOGIQ View) was then performed by scanning from distal to proximal, terminating at the myotendinous junction of the soleus to generate a full-length tendon image. Power Doppler imaging was subsequently performed with the gain set to 14, and a long axis cineloop was acquired at the tendon midpoint. Additional Doppler images were captured when vascularity was present; no Doppler images were obtained when vascularity was absent. See Figure 3 for details.

Patellar tendon imaging was performed using a FORTIS ultrasound system equipped with a 6–15 L linear transducer. Imaging parameters were standardized across all participants with a depth of 2.5 cm, frequency of 15 MHz, and gain of 56. Participants were seated upright with the back supported against a table or wall. A foam roller was placed beneath the knees to achieve 30 knee flexion, verified using a goniometer aligned from the greater trochanter to the lateral malleolus. The foot was supported as needed. The distal apex of the patella, the proximal deep tibial attachment of the patellar tendon, and the midpoint between these two locations were identified using the ultrasound center line and marked with a permanent marker. Imaging of the right side was performed first, followed by the left, using a standardized bilateral sequence. Short-axis views were obtained with one image captured 0.5 cm below the apex of the patella and a second image captured 0.5 cm above the proximal tibial attachment, both recorded as cineloops. A panoramic LOGIQ View scan was then performed in the proximal-to-distal direction, with a full-length tendon image (P1) acquired using the same 0.5 cm offsets. Finally, power Doppler imaging (PDI) was performed with long-axis views obtained at both the proximal and distal tendon insertions.

### 2.5. Ultrasound Imaging and Measurement Protocol

All measurements were performed using OsiriX MD, version 14.0 (Pixmeo SARL, Geneva, Switzerland) software to ensure consistency in post-processing and measurement tools across all sessions. For the Achilles tendon, CSA and thickness were measured at the intermalleolar midpoint, while vascularity was assessed using PDI and echogenicity was evaluated using B-mode ultrasound. For the patellar tendon, CSA and thickness were measured at three levels: the patellar apex, midpoint, and proximal tibial attachment. Patellar tendon vascularity was assessed using PDI at the proximal and distal insertions, echogenicity was evaluated using B-mode imaging, and total tendon length was also recorded.

#### Reliability

Measurement reliability of our researchers was excellent, with an intraclass correlation coefficient (ICC_3,1_ = 0.97).

### 2.6. Statistical Analysis

Analyses were conducted using R (R Foundation for Statistical Computing, Vienna, Austria, version 4.3.0). An alpha level of 0.05 was set to determine statistical significance. Continuous ultrasound outcomes were summarized as mean ± SD. For season-long comparisons across Summer, Preseason, and Postseason, within-subject pairwise differences were evaluated using paired-samples *t*-tests, with Wilcoxon signed-rank tests used as sensitivity analyses when normality assumptions were not met. To control the family-wise error rate across pairwise comparisons within each outcome, *p*-values were adjusted using the Holm method. Acute loading effects at Postseason were assessed using paired *t*-tests or Wilcoxon signed-rank tests, as appropriate. Effect sizes were calculated for pairwise comparisons using Cohen’s d for paired data, and 95% confidence intervals were reported where applicable. Statistical significance was set a priori at *p* < 0.05 (two-tailed).

Although paired comparisons were used to evaluate differences between timepoints, repeated-measures ANOVA or linear mixed-effects models would more appropriately account for within-subject correlation and unequal data structure. Therefore, the paired-test approach should be interpreted as a limitation of the current analysis.

To evaluate robustness of conclusions, prevalence patterns were re-examined using complete-case data only; interpretation of seasonal pain trends was unchanged. Demographic data were collected using Qualtrics (XM Platform 2024–2025, Provo, UT, USA; 2019), and imaging was performed after training sessions. Athletes were not excluded on the basis of current injury or rehabilitation status in order to characterize tendon structural characteristics across the full spectrum of training exposure representative of a real-world collegiate gymnastics team.

## 3. Results

A total of twenty-five Division I female gymnasts (age: 20.0 ± 1.6 years, weight: 57.8 ± 5.7 kg, height: 159.5 ± 6.2 cm, years of experience: 15.6 ± 1.7 y) self-selected their preferred leg (PL), their push-off or lead leg during jumping and tumbling skills. This information was obtained through verbal questioning of participants during the initial intake visit.

### 3.1. Seasonal Adaptation of Achilles Tendon

The morphology of the Achilles tendon demonstrated selective and significant adaptation across the competitive season. Right Achilles tendon thickness increased from Summer to Postseason (+5.8%, Cohen’s d = 0.65, 95% confidence interval (CI) 0.20–1.09, *p* = 0.0045, adjusted *p* = 0.0125), indicating a moderate effect size. No significant differences were observed between Summer to Preseason or Preseason to Postseason. The magnitude of this change suggests a small-to-moderate effect, although effect sizes were not formally calculated.

**Figure 4:**
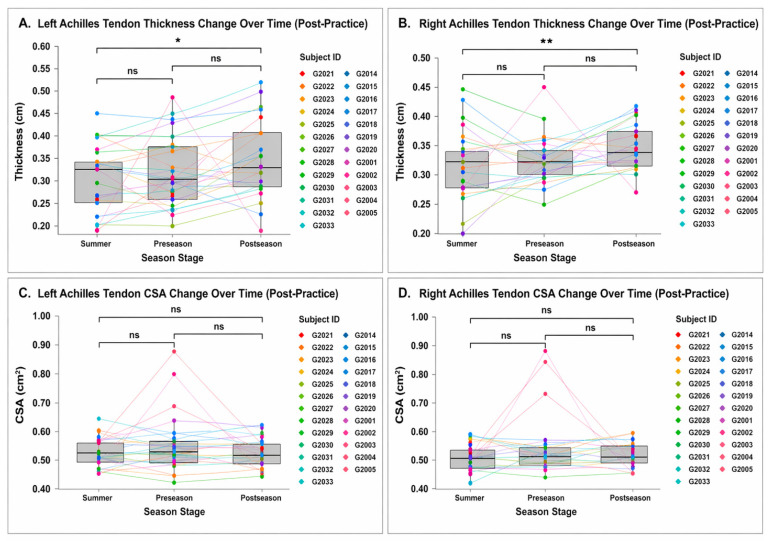
Achilles tendon thickness and cross-sectional area across the competitive season (post-practice). (**A**) Left Achilles tendon thickness; (**B**) right Achilles tendon thickness; (**C**) left Achilles tendon cross-sectional area; (**D**) right Achilles tendon cross-sectional area. Data are presented as individual values connected across timepoints, with boxplots representing median and interquartile range at each timepoint. Statistical comparisons were performed across seasonal stages. Abbreviations: CSA = cross-sectional area; cm = centimeters; cm^2^ = square centimeters; Symbols: ns = not statistically significant; * = *p* < 0.05; ** = *p* < 0.01.

In contrast, Achilles CSA did not exhibit consistent or widespread changes across the season; only one right-sided CSA comparison demonstrated a significant unadjusted effect (+4.4%, *p* = 0.019), which did not remain significant following adjustment (adjusted *p* = 0.056). Figure 4 is intended to display the individual longitudinal patterns underlying the statistical comparisons in Table 1, with the primary statistically significant finding being the increase in right Achilles tendon thickness from Summer to Postseason after Holm correction. See Table 1 and Figure 4.

No significant changes in Achilles tendon echogenicity or power Doppler signal were observed across the season (all *p* > 0.05), indicating that increased tendon thickness occurred in the absence of detectable alterations in vascularity.

### 3.2. Seasonal Adaptation of Patellar Tendon

**Figure 5:**
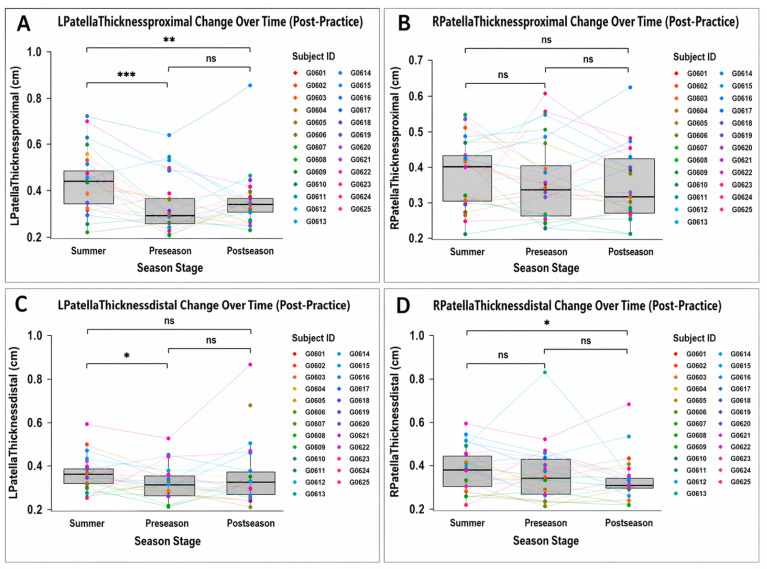
Patellar tendon thickness at proximal and distal regions across the competitive season (post-practice). (**A**) Left proximal patellar tendon thickness; (**B**) right proximal patellar tendon thickness; (**C**) left distal patellar tendon thickness; (**D**) right distal patellar tendon thickness. Data are presented as individual values connected across timepoints, with boxplots representing the distribution at each timepoint (median and interquartile range). Statistical comparisons were performed across seasonal stages. Abbreviations: L = left; R = right; cm = centimeters;. Symbols: ns = not statistically significant; * = *p* < 0.05; ** = *p* < 0.01; *** = *p* < 0.001.

Patellar tendon morphology remained largely unchanged across all three measured timepoints. No patellar CSA regions demonstrated significant season-long changes. However, left proximal patellar tendon thickness decreased from Summer to Preseason (−20.9%, Cohen’s d = 0.84, 95% CI 0.36–1.31, *p* = 0.0005, adjusted *p* = 0.002), indicating a large effect size. Thickness remained lower at Postseason compared with Summer (Cohen’s d = 0.71, 95% CI 0.27–1.15, *p* = 0.002, adjusted *p* = 0.005), indicating a moderate-to-large effect size, with no significant difference between Preseason and Postseason. No consistent alterations were detected in the proximal, mid-, or distal tendon regions. Figure 5 illustrates individual tendon trajectories, with statistically supported findings limited to the left proximal region. See Table 2 and Figure 5.

### 3.3. Seasonal Adaptation of Pain

Pain was assessed using the Visual Analog Scale (VAS) following training sessions and converted into binary indicators of pain presence or absence for descriptive reporting. Missing pain responses were treated as missing data and were not coded as absence of pain. Complete-case summaries were used for pain prevalence estimates to avoid introducing systematic bias from unreported responses. Pain prevalence increased modestly across the competitive season for both Achilles and patellar tendons, with no clear lateralized pattern. Because pain outcomes were binary and the sample size was limited, pain analyses were interpreted descriptively; however, logistic regression or mixed-effects logistic models would provide a more rigorous approach in future studies. See Table 3.

Formal statistical associations between side-to-side symmetry indices and pain could not be evaluated due to the binary nature of the pain data and absence of concurrent quantitative pain intensity measures. Descriptively, athletes with pain were observed across a wide range of symmetry values, suggesting no obvious threshold relationship between asymmetry and pain presence.

## 4. Discussion

The primary objective of this study was to evaluate seasonal changes in Achilles and patellar tendon morphology in collegiate gymnasts, while a secondary objective was to examine relationships with pain. Achilles tendon thickness increased throughout the competitive season, whereas Achilles CSA and patellar tendon morphology remained largely unchanged. These findings suggest tendon-specific morphological responses to the demands of collegiate gymnastics, consistent with prior work indicating that tendons may remodel differently depending on their strain environment and functional role [1,2,3,5].

The increase in Achilles tendon thickness occurred without detectable changes in echogenicity or power Doppler signal. This pattern may be suggestive of load-related structural response rather than overt pathological remodeling; however, the present study cannot confirm tendon health or mechanical adaptation because key indicators such as tendon stiffness, elasticity, functional performance, and direct workload metrics were not measured [15,17,18,19,20,21,22,23,24]. Therefore, these findings should be interpreted as being consistent with a possible adaptive response, rather than as definitive evidence of non-pathological remodeling. This distinction is important because tendon morphology alone does not necessarily reflect mechanical properties or clinical status, and ultrasound findings may not consistently correspond with pain in athletic populations [10,11,12,13,14,25].

In contrast, patellar tendon morphology remained relatively stable across the season, with the exception of localized reductions in left proximal thickness. These findings may reflect differences in cumulative loading patterns, tendon-specific mechanical demands, or regional sensitivity to adaptation; however, interpretation is limited by the absence of direct workload quantification. Because event-specific exposure, landing frequency, and jump volume were not measured, tendon-specific differences should be interpreted cautiously.

Ultrasound-based tendon monitoring shows promise as a practical tool for characterizing tendon-specific responses to high-impact training in collegiate gymnastics and for identifying athletes at increased risk of time-loss from injury [26,27]. While imaging options like magnetic resonance imaging are available, ultrasound is a more cost-effective, accessible, and dynamic tool that enables real-time evaluation of tendon structure, making it well suited for longitudinal monitoring in athletes.

Establishing preseason baselines and tracking thickness across the season may help identify athletes who exhibit atypical or disproportionate Achilles changes, allowing clinicians to recognize potential early overload before symptoms emerge [7,14,17,26]. Because vascular changes are not consistently present even in symptomatic tendons, monitoring structural measures such as thickness may provide more practical insight into load-related tendon response in athletic populations. This approach aligns with contemporary load-management frameworks that emphasize early detection of excessive or insufficient adaptation to training stimulus [2,4].

Regular ultrasound assessments can provide valuable structural information that complements subjective symptom reporting and performance metrics. The high reliability of Achilles ultrasonography, when performed by trained clinicians [14], supports its usefulness for routine monitoring in athletic populations. Although no changes in patellar tendon morphology occurred across the season, gymnastics inherently loads the lower limbs asymmetrically through skills such as twisting, vault approaches, and single-leg landings. Together, these findings indicate minimal in-season patellar tendon remodeling, suggesting either lower cumulative strain or greater morphological resilience relative to the Achilles tendon [12,22,23,24,25]. Previous epidemiological work demonstrates that limb-dominant loading patterns influence injury risk in women’s collegiate gymnastics [1,2,3,4]. Assessing side-to-side structural variation, even when morphology does not change, may help detect athletes who accumulate uneven mechanical load across limbs [8].

Limitations include the single-team convenience sample, absence of direct workload quantification (e.g., jump counts, landings), and reliance on self-reported pain measures. The lack of workload data limits interpretation of the relationship between mechanical loading and tendon adaptation. Additionally, although efforts were made to address missing data appropriately, incomplete responses may have influenced pain prevalence estimates.

Imaging was performed by a single examiner to reduce variability, but ultrasound acquisition remains operator-dependent [13]. The absence of elastography prevented assessment of tendon stiffness and mechanical properties, which are important complementary indicators of tendon health and may detect pathological change earlier than morphology alone [18,19,20,21,24]. Pain data were also limited by incomplete responses and binary classification. Missing pain responses were treated as missing rather than coded as no pain; nevertheless, incomplete pain reporting may have influenced prevalence estimates and limited the ability to model associations between pain and tendon morphology. Future studies incorporating concurrent quantitative pain measures, complete symptom tracking, workload metrics, and symmetry indices will be necessary to clarify whether side-to-side morphological differences meaningfully relate to pain in collegiate gymnasts.

The observed tendon-specific adaptation patterns reinforce the value of longitudinal ultrasound for screening and workload management in high-impact sports. Establishing preseason structural baselines enables clinicians to detect deviations during the competitive season, particularly changes in Achilles thickness that may reflect increased mechanical demand. Return-to-play decisions may benefit from incorporating structural normalization especially restoration of expected thickness patterns and preserved echogenicity alongside symptom resolution and functional capacity [28].

Overall, these findings support the integration of ultrasound imaging into preseason screening, in-season monitoring, and return-to-play decision-making frameworks within women’s collegiate gymnastics to inform decision-making. They reinforce the relevance of functional morphology-based monitoring strategies for athlete management, supporting the emphasis on integrative approaches linking structure, function, and performance [28].

## 5. Conclusions

This study demonstrates that Achilles tendon thickness increases across a competitive season in female collegiate gymnasts, while patellar tendon morphology remains stable. These findings are consistent with load-related adaptation and highlight the potential value of longitudinal ultrasound monitoring for athlete screening and load management. Monitoring these changes may support early identification of excessive loading and guide injury prevention strategies.

These results provide preliminary insight into how different lower-extremity tendons respond to sustained high-impact demands in collegiate gymnastics and support the clinical relevance of monitoring Achilles tendon structure across the competitive season. Tracking tendon thickness over time may help identify athletes exhibiting disproportionate adaptation and inform individualized load management strategies for future gymnasts.

Future research should expand these observations across multiple collegiate programs and seasons and integrate complementary measures such as elastography, biomechanical load quantification, and functional performance testing to better define the relationship between tendon remodeling, performance or injury risk.

## Figures and Tables

**Table 1:** Achilles tendon thickness and Cross-Sectional area across timepoints with Pairwise Comparisons (*n* = 25). Data are presented as mean ± standard deviation. Statistical significance was set at *p* < 0.05. Adjusted *p*-values were calculated using the Holm correction method.

Variable	Summer (Mean ± SD)	Preseason (Mean ± SD)	Postseason (Mean ± SD)	Comparison	t (df)	*p*	*p*_adj
Right Thickness (cm)	0.3586 ± 0.049	0.3636 ± 0.034	0.3793 ± 0.033	Summer vs. Preseason	−0.49 (24)	0.632	1.000
				Summer vs. Postseason	−3.19 (23)	0.0045	0.0125 *
				Preseason vs. Postseason	−1.87 (23)	0.075	0.224
Left Thickness (cm)	0.360 ± 0.032	0.364 ± 0.033	0.376 ± 0.037	Summer vs. Preseason	−0.65 (24)	0.527	1.000
				Summer vs. Postseason	−2.43 (23)	0.024	0.071
				Preseason vs. Postseason	−1.85 (23)	0.090	0.268
Right CSA (cm^2^)	0.410 ± 0.065	0.465 ± 0.170	0.428 ± 0.062	Summer vs. Preseason	−1.66 (22)	0.111	0.333
				Summer vs. Postseason	−2.57 (21)	0.019	0.056
				Preseason vs. Postseason	1.02 (23)	0.321	0.962
Left CSA (cm^2^)	0.403 ± 0.056	0.440 ± 0.124	0.403 ± 0.059	Summer vs. Preseason	−1.56 (24)	0.134	0.401
				Summer vs. Postseason	−0.16 (23)	0.824	1.000
				Preseason vs. Postseason	1.60 (23)	0.123	0.369

Abbreviations: CSA = Cross-sectional area; SD = standard deviation; *p*_adj = adjusted *p*-value. Symbols: * = statistically significant after adjustment.

**Table 2:** Patellar tendon thickness across timepoints with pairwise comparisons (*n* = 25). Data are presented as mean ± standard deviation. Statistical significance was set at *p* < 0.05 with Holm correction applied for multiple comparisons.

Variable	Summer (Mean ± SD)	Preseason (Mean ± SD)	Postseason (Mean ± SD)	Comparison	t (df)	*p*	*p*_adj
Left Proximal Thickness (cm)	0.404 ± 0.088	0.394 ± 0.104	0.386 ± 0.099	Summer vs. Preseason	4.04 (22)	0.0005	0.002 *
				Summer vs. Postseason	3.56 (24)	0.002	0.005 *
				Preseason vs. Postseason	−0.61 (22)	0.551	1.000
Right Proximal Thickness (cm)	0.377 ± 0.096	0.362 ± 0.132	0.336 ± 0.093	Summer vs. Preseason	0.384 (23)	0.704	1.000
				Summer vs. Postseason	0.863 (24)	0.397	1.000
				Preseason vs. Postseason	0.654 (23)	0.520	1.000
Left Distal Thickness (cm)	0.446 ± 0.119	0.353 ± 0.114	0.371 ± 0.114	Summer vs. Preseason	2.58 (22)	0.017	0.051
				Summer vs. Postseason	0.20 (24)	0.845	1.000
				Preseason vs. Postseason	−1.42 (22)	0.171	0.513
Right Distal Thickness (cm)	0.362 ± 0.071	0.320 ± 0.070	0.357 ± 0.139	Summer vs. Preseason	0.739 (23)	0.467	1.000
				Summer vs. Postseason	2.189 (24)	0.038	0.115
				Preseason vs. Postseason	0.926 (23)	0.364	1.000

Abbreviations: CSA = Cross-sectional area; SD = standard deviation; *p*_adj = adjusted *p*-value. Symbols: * = statistically significant after adjustment.

**Table 3:** Prevalence of lower-extremity pain across gymnastics season (*n* = 25). Data are presented as frequency (percentage).

Pain Location and Duration	Summer 2024 *n* (%)	December 2024 *n* (%)	April 2025 *n* (%)	Percentage Point Change (Summer to April)
Right Achilles pain (past 12 months)	3 (12%)	5 (20%)	6 (24%)	+12 pp
Right Achilles pain (≥1 year)	7 (28%)	10 (40%)	10 (40%)	+12 pp
Left Achilles pain (past 12 months)	3 (12%)	8 (32%)	9 (36%)	+24 pp
Left Achilles pain (≥1 year)	5 (20%)	6 (24%)	8 (32%)	+12 pp
Right patellar pain (past 12 months)	5 (20%)	5 (20%)	7 (28%)	+8 pp
Right patellar pain (≥1 year)	6 (24%)	8 (32%)	10 (40%)	+16 pp
Left patellar pain (past 12 months)	3 (12%)	3 (12%)	4 (16%)	+4 pp
Left patellar pain (≥1 year)	6 (24%)	6 (24%)	6 (24%)	0 pp

Abbreviations: pp = percentage points.

## Data Availability

All images obtained from the ultrasound scans were loaded into OsiriX (Pixmeo SARL, Geneva, Switerland) in order to obtain measurements. The data presented in this study are available on request from the corresponding author due to privacy restrictions.

## Abbreviations

The following abbreviations are used in this manuscript:

CSA	Cross-Sectional Area
GenAI	Generative Artificial Intelligence
ICC	Intraclass Correlation Coefficient
NCAA	National Collegiate Athletic Association
PDI	Power Doppler imaging
PL	Preferred Leg
PP	Percentage Point
SOP	Standard Operating Procedure
VAS	Visual Analog Scale

## References

[1] Kobayashi J.K., Kobayashi E.F., Tomasevich K.M., Lorens K.L., Aoki S.K. (2023). Characterization of Achilles tendon ruptures in collegiate women’s gymnastics. Orthopedics.

[2] Fryar C., Tilley D., Casey E., Vincent H.K. (2023). A research and clinical framework for understanding Achilles injury in female collegiate gymnasts. Curr. Sports Med. Rep..

[3] Bonanno J., Cheng J., Tilley D., Abutalib Z., Casey E. (2022). Factors associated with Achilles tendon rupture in women’s collegiate gymnastics. Sports Health.

[4] Kerr Z.Y., Hayden R., Barr M., Klossner D.A., Dompier T.P. (2015). Epidemiology of National Collegiate Athletic Association women’s gymnastics injuries, 2009–2010 through 2013–2014. J. Athl. Train..

[5] Cook J.L., Purdam C.R. (2009). Is tendon pathology a continuum? A pathology model to explain the clinical presentation of load-induced tendinopathy. Br. J. Sports Med..

[6] Gabbett T.J. (2016). The training–injury prevention paradox: Should athletes be training smarter and harder?. Br. J. Sports Med..

[7] Steinberg N., Elbaz L., Nemet D., Ayalon M., Eliakim A. (2024). Tendon structure, clinical tests, and pain during loading in young female competitive gymnasts. J. Sports Sci..

[8] Theobald P., Benjamin M., Nokes L., Pugh N. (2005). Review of the vascularisation of the human Achilles tendon. Injury.

[9] Docking S.I., Cook J. (2016). Pathological tendons maintain sufficient aligned fibrillar structure on ultrasound imaging. J. Sci. Med. Sport.

[10] Emerson C., Morrissey D., Perry M., Jalan R. (2010). Ultrasonographically detected changes in Achilles tendons and self-reported symptoms in elite gymnasts compared with controls—An observational study. Man. Ther..

[11] Comin J., Cook J.L., Malliaras P., McCormack M., Calleja M., Clarke A., Connell D.A. (2013). The prevalence and clinical significance of sonographic tendon abnormalities in asymptomatic ballet dancers: A 24-month longitudinal study. Br. J. Sports Med..

[12] Kulig K., Oki K.C., Chang Y.-J., Bashford G.R. (2014). Achilles and patellar tendon morphology in dancers with and without tendon pain. Med. Probl. Perform. Art..

[13] Anker-Petersen C., Juul-Kristensen B., Antflick J., Aagaard H., Myers C., Boesen A.P., Boyle E., Hölmich P., Thorborg K. (2021). Six weeks of intensive rehearsals for Swan Lake ballet shows ultrasound tissue characterization changes of the Achilles tendons in dancers. Scand. J. Med. Sci. Sports.

[14] Pentidis N., Fersman S., Bohm S. (2021). Development of muscle–tendon adaptation in preadolescent gymnasts and untrained peers: A 12-month longitudinal study. Med. Sci. Sports Exerc..

[15] Cassel M., Intziegianni K., Risch L., Müller J. (2017). Physiological tendon thickness adaptation in adolescent elite athletes: A longitudinal study. Front. Physiol..

[16] Steinberg N., Elbaz L., Nemet D., Eliakim A. (2025). Exploration of clinical diagnosis for tendinopathy, tendon structure, and muscle strength in young elite female gymnasts: A 12-month follow-up study. J. Sports Sci..

[17] Pelea M.A., Serban O., Badarinza M., Gutiu R., Fodor D. (2024). Shear-wave elastography of the Achilles tendon: Reliability analysis and impact of parameters modulating elasticity values. J. Ultrasound.

[18] Zhou J., Lin Y., Zhang J., Si’tu X., Wang J., Pan W., Wang Y. (2022). Reliability of shear wave elastography for the assessment of gastrocnemius fascia elasticity in healthy individuals. Sci. Rep..

[19] Ito N., Sigurðsson H.B., Pohlig R.T., Cortes D.H., Grävare Silbernagel K., Sprague A.L. (2022). Reliability of continuous shear wave elastography in the pathological patellar tendon. J. Ultrasound Med..

[20] Mifsud T., McWilliams D., Roberts H., Singh D. (2023). Elastography in the assessment of the Achilles tendon: Systematic review. J. Foot Ankle Res..

[21] Römer C., Zessin E., Czupajllo J., Fischer T., Wolfarth B., Lerchbaumer M.H. (2023). Effect of physical parameters and training load on patellar tendon stiffness in professional athletes. Diagnostics.

[22] Kongsgaard M., Qvortrup K., Larsen J., Aagaard P., Doessing S., Hansen P., Kjaer M., Magnusson S.P. (2010). Fibril morphology and tendon mechanical properties in patellar tendinopathy: Effects of heavy slow resistance training. Am. J. Sports Med..

[23] Kongsgaard M., Langberg H. (2011). Tendinopathy: Present challenges and perspectives for progression. Scand. J. Med. Sci. Sports.

[24] Mannarino F., de Matta T.T., de Oliveira L.F. (2019). An 8-week resistance training protocol is effective in adapting vastus lateralis muscle, but not patellar tendon shear modulus measured by shear-wave elastography. PLoS ONE.

[25] Notarnicola A., Maccagnano G., Di Leo G., Tafuri S., Moretti B. (2014). Overload and neovascularization of Achilles tendons in young artistic and rhythmic gymnasts compared with controls: An observational study. Musculoskelet. Surg..

[26] Cushman D.M., Stokes D., Vu L., Corcoran B., Fredericson M., Eby S.F., Teramoto M. (2025). Ultrasound as a predictor of time-loss injury for the patellar tendon, Achilles tendon and plantar fascia in Division I collegiate athletes. Br. J. Sports Med..

[27] Paantjens M., van der Leeuw M., Helmhout P., Isaac A., De Maeseneer M. (2020). The interrater reliability of ultrasonography for Achilles tendon structure. J. Ultrason..

[28] McAuliffe S., McCreesh K., Culloty F., Purtill H., O’Sullivan K. (2016). Can ultrasound imaging predict the development of Achilles and patellar tendinopathy? A systematic review and meta-analysis. Br. J. Sports Med..

